# Griseofulvin impairs intraerythrocytic growth of *Plasmodium falciparum* through ferrochelatase inhibition but lacks activity in an experimental human infection study

**DOI:** 10.1038/srep41975

**Published:** 2017-02-08

**Authors:** Clare M. Smith, Ante Jerkovic, Thy Thuc Truong, Simon J. Foote, James S. McCarthy, Brendan J. McMorran

**Affiliations:** 1 School of Medicine and The Menzies Research Institute, University of Tasmania, Hobart, Tasmania, Australia; 2 Department of Biomedical Sciences, Faculty of Medicine and Health Sciences, Macquarie University, Sydney, New South Wales, Australia; 3 Joint Mass Spectrometry Facility, Research School of Chemistry, The Australian National University, Canberra, Australian Capital Territory, Australia; 4 The John Curtin School of Medical Research, The Australian National University, Canberra, Australian Capital Territory, Australia; 5 QIMR Berghofer Medical Research Institute, Brisbane, Queensland, Australia

## Abstract

Griseofulvin, an orally active antifungal drug used to treat dermatophyte infections, has a secondary effect of inducing cytochrome P450-mediated production of *N*-methyl protoporphyrin IX (*N*-MPP). *N-*MPP is a potent competitive inhibitor of the heme biosynthetic-enzyme ferrochelatase, and inhibits the growth of cultured erythrocyte stage *Plasmodium falciparum*. Novel drugs against *Plasmodium* are needed to achieve malaria elimination. Thus, we investigated whether griseofulvin shows anti-plasmodial activity. We observed that the intraerythrocytic growth of *P. falciparum* is inhibited in red blood cells pretreated with griseofulvin *in vitro*. Treatment with 100 μM griseofulvin was sufficient to prevent parasite growth and induce the production of *N*-MPP. Inclusion of the ferrochelatase substrate PPIX blocked the inhibitory activity of griseofulvin, suggesting that griseofulvin exerts its activity through the *N*-MPP-dependent inhibition of ferrochelatase. In an *ex-vivo* study, red blood cells from griseofulvin-treated subjects were refractory to the growth of cultured *P. falciparum*. However, in a clinical trial griseofulvin failed to show either therapeutic or prophylactic effect in subjects infected with blood stage *P. falciparum*. Although the development of griseofulvin as an antimalarial is not warranted, it represents a novel inhibitor of *P. falciparum* growth and acts via the *N*-MPP-dependent inhibition of ferrochelatase.

Each year *Plasmodium falciparum* malaria affects over 200 million people and approximately 300,000 children succumb to the disease^1^. Successful treatment, and ultimately elimination of malaria requires effective antimalarial drugs. Our current armamentarium is threatened by the development and spread of drug resistant strains of the parasite, with key frontline artemisinin combination therapies showing failure in the Greater Mekong Subregion^2^. New drugs that act on novel targets are urgently required.

A relatively unexplored approach to identify new targets for antimalarials includes host enzymes co-opted by *Plasmodium*^3^. Several red blood cell (RBC) factors are scavenged by the parasite during intra-erythrocytic growth, including redox enzymes, protein kinases and biosynthetic enzymes^4^,^5^,^6^,^7^. One of these enzymes, ferrochelatase (FECH; EC 4.99.1.1), catalyzes the final step in the biosynthesis of heme, incorporating ferrous iron into the protoporphyrin IX (PPIX) ring. *N*-methylprotoporhyrin IX (*N*-MPP), an *N*-alkyl porphyrin and a potent competitive inhibitor of FECH, prevents the *in vitro* growth of *P. falciparum,* indicating that FECH activity is essential to the parasite^8^. However, knockout studies of both *P. falciparum* and the murine parasite, *P. berghei*, indicate that parasite FECH is dispensable for the erythrocytic stage of the lifecycle^9^,^10^. We recently demonstrated the host FECH is necessary for the parasite to grow and cause malaria infection^8^. This finding is supported by observations that *Plasmodium* contains intact and active human FECH^11^. Thus, drugs that inhibit host FECH may prevent parasite growth.

The clinically approved antifungal drug griseofulvin is a known FECH inhibitor. Griseofulvin has been used to treat human dermatophyte fungal infections for over 50 years. It accumulates in fungi and binds to microtubules; the accumulation of griseofulvin in keratin precursor cells renders them resistant to fungal infection^12^. A second pharmacologic effect of griseofulvin is inhibition of FECH. This manifests in humans with porphyria and in mice as acute attacks of porphyria^13^,^14^. In the mouse liver, griseofulvin is metabolized by cytochrome P450 enzymes to produce griseofulvin-PPIX adducts and *N*-MPP; *N*-MPP inhibits FECH and causes the accumulation of hepatotoxic heme precursors^15^. Similar toxicity is observed in humans with congenital deficiencies in heme biosynthetic enzymes^16^, although the drug is well-tolerated in healthy individuals.

Here we investigated if griseofulvin could inhibit the growth of cultured blood-stage *P. falciparum*, hypothesizing that griseofulvin would inhibit parasite growth via generation of the FECH inhibitor *N-*MPP. Secondly, we investigated if RBCs from griseofulvin-treated subjects reduced parasite growth. Thirdly, we investigated the ability of griseofulvin as treatment for malaria infection in an experimental human infection study.

## Results

### *P. falciparum in vitro* growth inhibition

**Figure 1:**
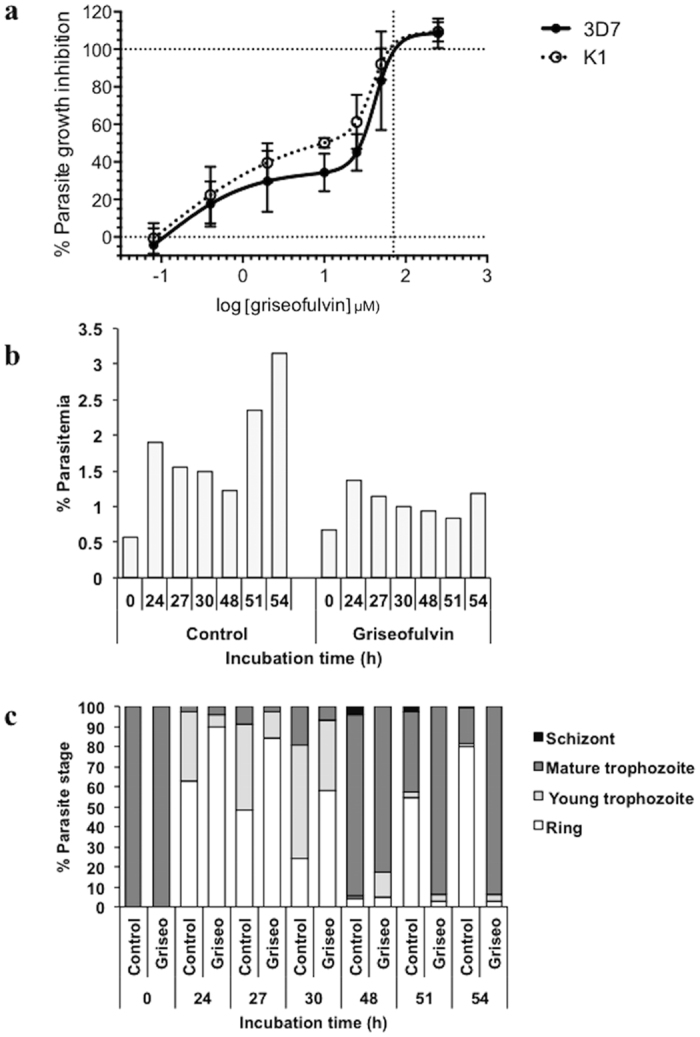
The effects of *in vitro* griseofulvin treatment on *P. falciparum* growth. (**a**) Inhibition of *P. falciparum* growth in griseofulvin-treated red blood cells, determined using the *in vitro* parasite growth assay. Red cells were incubated in the indicated concentrations of griseofulvin for three days prior to parasite inoculation and cultured for 54 h. Assays were conducted using *P. falciparum* strains 3D7 and K1. Data represent the mean (SD) of two independent assays (each concentration assayed in triplicate). Growth (**b**) and development (**c**) of *P. falciparum* 3D7 parasites in griseofulvin-treated red cells sampled at different times. Red blood cells were incubated in 50 μM griseofulvin for three days, and then inoculated with purified trophozoites. Total % parasitaemia and relative proportions of four parasite stages were determined just after inoculation (0 h) and at the indicated times. Assays were performed once for each time point.

In preliminary investigations we observed that the growth of *P. falciparum* in RBCs was not inhibited by griseofulvin if the compound was added directly to the parasite cultures. Subsequently, we observed that griseofulvin accumulates in RBCs during prolonged incubation, with maximal levels observed after three days (Supplementary Fig. S1). To test the anti-plasmodial activity of griseofulvin, we pretreated RBCs with griseofulvin for three days before inoculating with *P. falciparum*. We tested two different *P. falciparum* strains, 3D7 (chloroquine susceptible) and K1 (chloroquine resistant). Both strains showed an equivalent concentration-dependent response to exposure to the compound. The inhibition curves had partially biphasic slopes, with an apparent plateau between 2 and 10 μM corresponding to 30–40% growth inhibition. The growth of both strains was inhibited by more than 80% with 50 μM of the compound and complete inhibition (IC_100_) occurred at concentrations a greater than 100 μM (Fig. 1a). We also determined the percentage parasitemia and proportions of different parasite developmental stages grown in RBCs treated with 50 μM griseofulvin (Fig. 1b and c). Following infection with trophozoite stage parasites, there was no obvious difference in percentage parasitemia between treated and control cultures after 24 h, indicating that production of first generation parasites was similar, and that griseofulvin treatment did not affect merozoite invasion and early growth in the RBC. However, there was a notable delay in the treated cultures of parasites transitioning from ring stage to young trophozoite, and then to mature pigmented trophozoites between 24–48 h, as well as an absence of schizonts (48–54 h). There was as well a lack of second-generation parasite production in the treated cultures in contrast to control parasites whose concentration doubled between 48–54 h, with a transition from mature trophozoites and schizonts to ring and young trophozoite stages. In contrast, the parasitemia did not change in the treated cultures between 48–54 h, and the majority of the parasites remained as pigmented trophozoites. This suggested that griseofulvin impaired the intraerythrocytic development of parasites.

**Figure 2:**
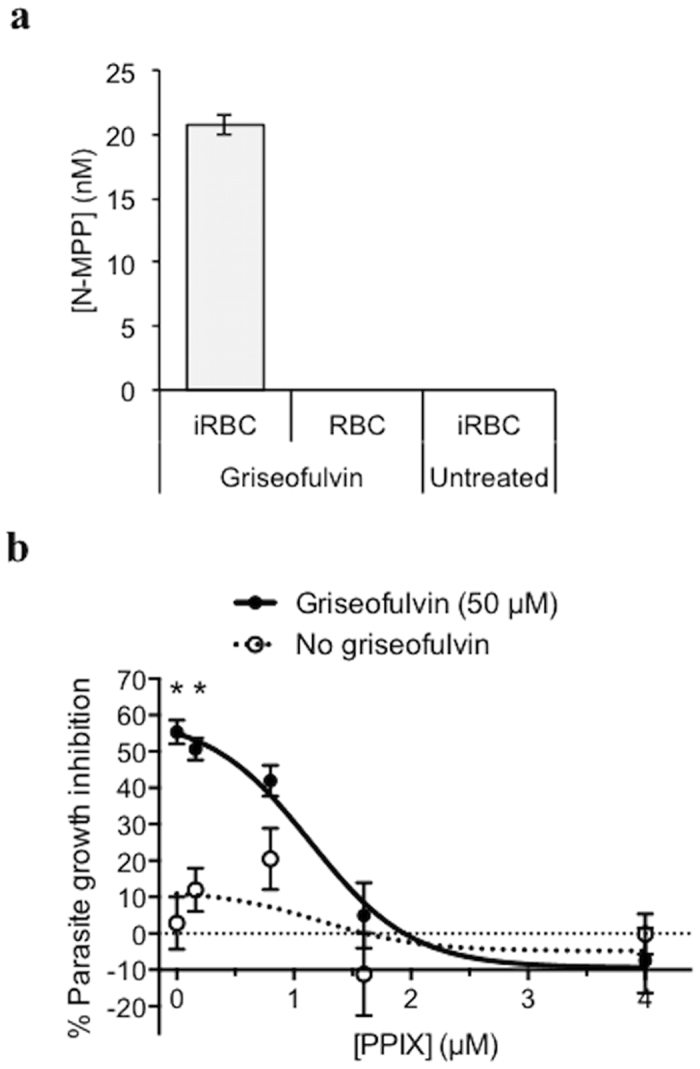
Analysis of the griseofulvin cytotoxic effect on *P. falciparum*. (**a**) *N*-MPP concentrations measured in *P. falciparum*-infected red blood cells (iRBC) and uninfected red blood cells (RBC) following treatment with griseofulvin treatment (100 μM), and in untreated iRBC. No *N-*MPP was detected in griseofulvin treated RBC or untreated iRBC. Data represent the mean (SD) of three independent assays. (**b**) Effect of different PPIX concentrations on the growth of *P. falciparum* incubated with griseofulvin (50 μM) or without griseofulvin (PPIX control). Assays were conducted for 54 h. Data represent mean (SEM) of two independent experiments (assayed in triplicate). *p < 0.001 by t-test and Bonferroni correction for multiple comparisons.

We hypothesized that the growth inhibitory effect of griseofulvin was via generation of the FECH inhibitor, *N-*MPP, which is cytotoxic against cultured *P. falciparum*^8^. Treatment of parasitized RBCs with 100 μM griseofulvin (approximately IC_100_ levels) resulted in detectable and quantifiable production of *N*-MPP. In contrast, no *N*-MPP was observed in drug-treated uninfected cells, or in untreated parasites cultured for the same period of time (Fig. 2a). To confirm the griseofulvin cytotoxicity was due to the *N-*MPP-dependent inhibition of FECH, we tested the addition of PPIX (FECH substrate), which blocks the parasite growth-inhibitory action of *N*-MPP^8^. Addition of 1.6 or 4 μM PPIX to parasites grown in 50 μM griseofulvin completely reversed the growth inhibitory activity of the drug; PPIX treatment alone had no effect on parasites at any of the concentrations tested (Fig. 2b and Table 1).

### Anti-malarial activity of griseofulvin in an *ex vivo* study

We tested if *P. falciparum* could grow in human RBCs from subjects taking a course of griseofulvin. Three subjects (E1, E2 and E3) took 1,000 mg/day of griseofulvin for four days (Table 2 and Supplementary Fig. S2). Blood samples were collected before they commenced treatment (control sample) and each day during the treatment, and their blood was tested to investigate whether it supported parasite growth. Treatment and control RBCs were infected with the same parasite inoculum and dose. After 17 h of culture, the numbers of parasitized cells were similar, but by 48 h parasite numbers were lower in treated versus control samples (Supplementary Fig. S3). This was indicative of a normally efficient first round of parasite invasion, but an impairment of the growth and propagation of second cycle parasites. The effect was apparent for samples collected each day from each subject during the treatment, and corresponded to an average daily percent growth inhibition ranging between 49–94% (CV% range: 19–51; Fig. 3a).

**Figure 3:**
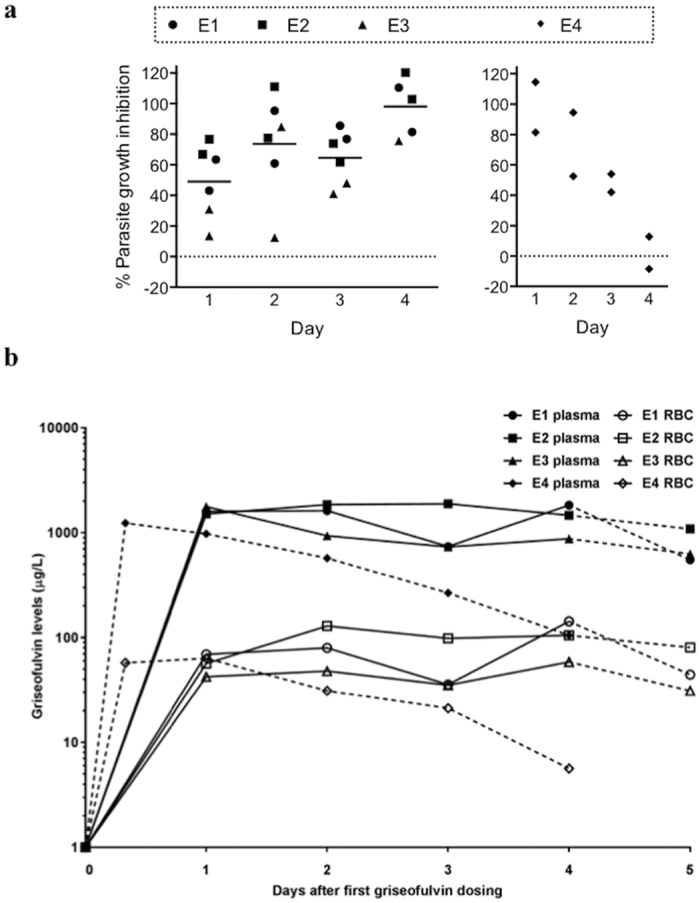
*P. falciparum* growth in red cells from griseofulvin-treated subjects and griseofulvin pharmacokinetics. (**a**) Percentage growth inhibition of *P. falciparum* cultured in red blood cells from griseofulvin-treated subjects. Parasite growth assays were performed on red blood cells collected from three subjects taking 1,000 mg griseofulvin/day (E1, E2 and E3) and one subject after a single 2,000 mg dose (E4). Parasites were cultured for 48 h. Data represent the percent growth inhibition determined in samples collected 1, 2, 3 and 4 days after initiating the treatment, and mean (horizontal line). Each sample was assayed twice. (**b**) Concentrations of griseofulvin in plasma and red blood cells of the same griseofulvin-treated subjects. Dashed lines indicate the time-point when dosing with griseofulvin ceased. Data represent the mean of duplicate assays.

A fourth subject, E4, took a single 2,000 mg dose of griseofulvin (Table 2). After dosing, samples were collected each day for four days to measure drug concentrations and to investigate parasite growth inhibition. Parasite growth in RBCs was markedly impeded on Day 1 and 2 post-treatment, with a parasite growth inhibition of 98% and 73%, respectively. Parasite growth inhibition decreased on Day 3 (48%), and was absent on Day 4 (Fig. 3a and Supplementary Fig. S3).

We measured levels of griseofulvin in plasma and in RBCs from the four subjects (Fig. 3b and Table 3). In Subjects E1-3, the level of griseofulvin in plasma was ~17 fold higher than that in RBCs. There was some inter-individual variation in the griseofulvin levels in RBCs (CV% = 40.9). The highest plasma level of griseofulvin in Subject E4 was observed 8 h after receiving the dose, and then progressively fell over the following days. As observed in the RBCs from subjects who had received repeated doses of griseofulvin, levels in plasma were higher than in RBCs (~20 fold). There were modest correlations between plasma and RBC levels in subjects taking 1,000 mg/day, but a more robust relationship was observed in the subject who received 2,000 mg single dose; plasma and RBC griseofulvin concentrations declined at the same rate in this subject. Griseofulvin was not detected, or detected at very low levels, 12 (E1, E2), 14 (E4) and 15 (E3) days after initial griseofulvin dosing.

### Anti-plasmodial activity of griseofulvin in an induced blood stage malaria (IBSM) clinical study

**Figure 4:**
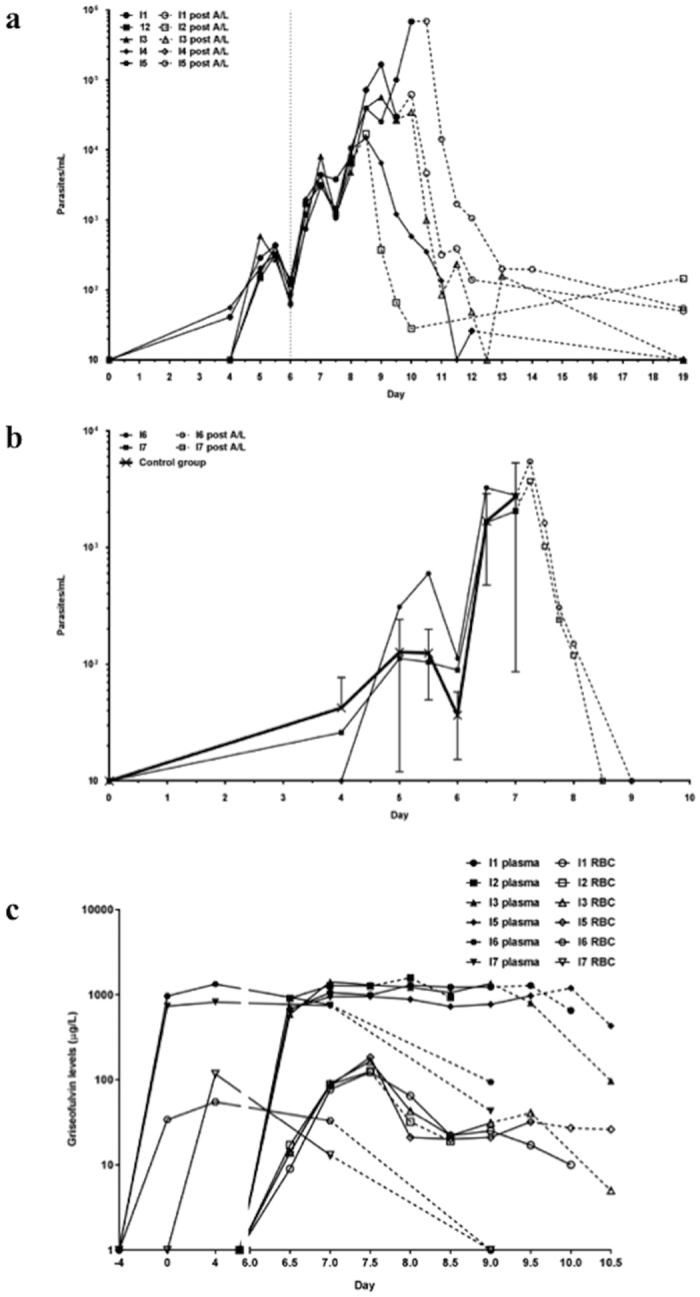
Parasite growth and griseofulvin pharmacokinetics in *P. falciparum*-infected subjects with griseofulvin. Two cohorts were experimentally infected with blood stage *P. falciparum* and monitored for parasitemia by PCR. Day 0 corresponds with inoculation day for both cohorts. In Cohort 1 (**a**), four subjects (I1, I2, I3 and I5) began taking griseofulvin (500 mg BD) on Day 6 after inoculation (vertical line). Griseofulvin dosing ended on different days (Table 1) depending on PCR parasitaemia quantification. Dashed lines indicate the time-point when participants received treatment with artemether-lumefantrine (A/L). Subject I4 was a control subject and received treatment with mefloquine on Day 8. In Cohort 2 (**b**), two subjects took griseofulvin (500 mg BD) from Day -4 (4 days before infection) until Day 6. Both subjects in Cohort 2 were treated with artemether-lumefantrine on Day 7. Commencement of treatment with artemether-lumefantrine (A/L) is indicated with dashed lines. An additional eight subjects that did not receive griseofulvin (control group) were infected at the same time with the same inoculum; mean parasite levels (SD) for this group are shown as a comparison. (**c**) Concentrations of griseofulvin in plasma and red blood cells of the same Cohort 1 and Cohort 2 *P. falciparum*-infected subjects. Dashed lines indicate the time-point when dosing with griseofulvin ceased and participants received treatment with artemether-lumefantrine. Day 0 corresponds with inoculation day. Data represent the mean of duplicate assays.

To determine if griseofulvin showed *in vivo* activity against *P. falciparum*, we performed a pilot clinical study in two cohorts. The first subject enrolled on August 20, 2013, and the last subject finished the study on March 11, 2015. From the 16 subjects screened, seven were enrolled in the study (Supplementary Fig. S4 and Supplementary Table S1). Cohort 1 was designed to test if griseofulvin showed clinically significant activity in blood stage malaria infection. All subjects in this cohort (n = 5) became infected and showed the expected parasite growth profile, with parasitemia becoming detectable by PCR on Day 4 (Fig. 4a). Four subjects in Cohort 1 (I1, I2, I3, and I5) began treatment with griseofulvin on Day 6 (500 mg BD), but the treatment showed no effect on parasite growth. Subjects received rescue treatment with artemether-lumefantrine at different time points as per protocol when their parasitemia curves demonstrated that griseofulvin did not show significant inhibition of parasite growth (Table 2). Subject I3 was admitted to the hospital on Day 9 to treat a serious adverse event unrelated to the study (renal colic). This subject was treated with I.V. artesunate (180 mg) on Day 9 *per protocol*. All subjects in the griseofulvin arm (n = 4) cleared parasitemia rapidly after treatment with artemether-lumefantrine. A control subject (n = 1), Subject I4, received treatment with mefloquine on Day 8, with parasitemia clearing by Day 11. This subject also received treatment with artemether-lumefantrine *per protocol* at the end of study (Fig. 4a and Table 2). Because no significant parasite clearance was observed in the griseofulvin treated subjects, the parasite reduction ratio (PRR) could not be determined. Parasitemia levels decreased rapidly in all subjects in the griseofulvin arm (n = 4) after treatment with artemether-lumefantrine. Parasitemia levels on Day 19 were below the limit of detection of the qPCR assay (10 parasites/mL) for Subject I3, whilst residual parasitemia levels were observed in subjects I1, I2 and I5 (Fig. 4A). The parasitemia detected in these three subjects was probably due to the presence of gametocytes in their blood.

To assess whether griseofulvin may have an inhibitory effect on parasite growth if administered as a prophylactic agent, subjects in Cohort 2 (n = 2, subjects I6 and I7) were given griseofulvin from Day -4 (4 days prior to inoculation) till Day 6 (Table 2). However, no effect on parasite growth was observed. Parasite growth was very similar to subjects in a control cohort (n = 8), enrolled in a parallel study in the same clinical unit. These subjects were simultaneously inoculated with the same inoculum (Fig. 4b). Subjects in the griseofulvin study received curative treatment with artemether-lumefantrine on Day 7, with parasitemia clearing by Day 9. Although it was planned to include six subjects in this cohort, since no prophylactic effect was observed in these two subjects, the safety review team decided that no more subjects should be enrolled in the study. As no meaningful inhibition of parasite growth rate could be discerned, the parasite multiplication rate (PMR) could not be estimated.

Plasma and RBC levels of griseofulvin were determined at designated time-points (Fig. 4c and Table 3). In Cohort 1, griseofulvin plasma levels were highest 2 days after beginning therapy (Day 8). The level of griseofulvin in RBC was highest after one day of treatment (Day 7 PM), then decreased on Day 8. Repeat measures and correcting for hemoglobin concentration produced the same result (peak griseofulvin levels 151 μg/L, range 127–183), excluding technical factors and variable RBC yields as a possible explanation. In Cohort 2, griseofulvin plasma and RBC levels were highest on Day 4, ie, 8 days after commencement of griseofulvin treatment. As observed in the *ex vivo* study, plasma levels were higher than in RBCs (~9 fold for Cohort 1 and ~13 fold for Cohort 2), with significant inter-individual variation in griseofulvin levels again observed, especially in RBC. When the level of griseofulvin among subjects receiving the 1,000 mg dose in the *ex vivo* study was compared to subjects in Cohorts 1 and 2 of the IBSM study, plasma levels were significantly higher in the *ex vivo* study, whilst RBC levels were not significantly different (Table 3).

Plasma PPIX concentrations were determined in Cohort 2 subjects (I6 and I7) on Day 7 (Supplementary Fig. S5). Mean concentrations (SD) were 0.08 μM (0.03 μM) and 0.12 μM (0.06 μM), respectively. No PPIX was detected in two untreated control plasma samples (Supplementary Fig. S5). From these data and the griseofulvin measures determined above, we estimated the PPIX:griseofulvin plasma ratios ranged between 1:18 and 1:27 (Table 1).

#### Adverse events

In the *ex vivo* study, no abnormalities were noted and subjects experienced no adverse events. In the IBSM study, all subjects except I7 reported at least one adverse event. The majority of the events (49/57) were of mild severity, and most (45/57) were attributed to malaria and were consistent with those reported in previous studies. The most common adverse events are listed in Supplementary Table S2. One adverse event (renal colic) was classified as serious; however, this event was determined not to be related to study treatments. All adverse events had resolved by the end of study.

## Discussion

Our results demonstrate that griseofulvin inhibits the intraerythrocytic development of *P. falciparum* in both chloroquine-sensitive and resistant strains of *P. falciparum*. For griseofulvin to inhibit *P. falciparum*, RBCs must be pretreated with the drug prior to parasite infection. We also observed that *N*-MPP is produced in griseofulvin-treated *P. falciparum* infected RBCs, but not in uninfected RBCs. This suggests that the parasite utilizes a metabolic process to degrade griseofulvin similar to the P450-degradative process observed in animals as mature erythrocytes do not possess these enzymes^17^. *N*-MPP is a substrate analogue and specific competitive inhibitor of FECH (K_*i*_ in the nanomolar range). We have previously shown that *N*-MPP inhibits the growth of *P. falciparum in vitro*, and that this activity is blocked by the FECH substrate, PPIX, thus confirming *N*-MPP specificity for FECH rather than other targets^8^. In the current study, the growth inhibitory activity of griseofulvin was similarly blocked by the addition of PPIX. Collectively, these data indicate that griseofulvin acts against the parasite by inducing the production of *N*-MPP, which then inhibits FECH. The partly biphasic shape of the *in vitro* growth inhibition curve suggests a certain level of complexity in the mechanism of action. One possibility is the counteractive effects of *N*-MPP and PPIX production, derived from griseofulvin metabolism and FECH inhibition, respectively. Alternatively, or in addition, it may reflect the differing sensitivities of parasite-expressed and host FECH to *N*-MPP, should both be involved in the mechanism of growth inhibition. Of note, treatment of cultured *P. falciparum* with *N*-MPP produces a similar biphasic response^8^.

We also found that RBCs collected from subjects who had been orally administered clinically used doses of griseofulvin accumulated the drug in levels sufficient to impair parasite growth. Pharmacokinetic profiling demonstrated that levels of griseofulvin in the plasma and RBCs peaked within one day of subjects taking the drug. The levels achieved in the RBC (59–143 μg/L) were comparable to the levels required in *in vitro*-treated cells to effect parasite growth inhibition −20 μM treatment resulted in 0.110 μM (39 μg/L) intracellular griseofulvin (Supplementary Fig. S1) and approximately 40% parasite growth inhibition (Fig. 1a). RBCs from a subject given a single 2,000 mg dose inhibited parasite growth for up to 2 days, after which parasite growth inhibition gradually declined, coincidental with the fall in plasma and RBC levels of griseofulvin.

However, the antimalarial activity of griseofulvin that was observed *in vitro* and *ex vivo* did not translate into clinical activity when tested in an experimental malaria infection system. We tested the antimalarial activity of griseofulvin in the IBSM model using two different griseofulvin regimens: a treatment (Cohort 1) and a prophylactic regimen (Cohort 2). In both cohorts, no evidence of inhibition of parasite growth was observed. The sample size required to determine an antimalarial effect is larger than that used in this study, typically eight subjects per study arm^18^. However, because preliminary data indicated a lack of effect and a meaningful endpoint was unlikely to be reached, the investigators decided not to enroll more subjects and to close the study. Of note, there was a distinct and unexplainable decrease in the levels of griseofulvin in RBC in Cohort 1 of the IBSM study between Day 7 and Day 8. An exhaustive PK analysis of griseofulvin was not conducted because its PK profile has already been documented^19^,^20^. Therefore, determination and comparison of total drug exposure for the *ex vivo* and IBSM studies was not possible. Griseofulvin plasma levels were significantly higher in the *ex vivo* than in the IBSM study, but RBC levels were comparable. Since our *in vitro* results suggest that griseofulvin is converted into *N*-MPP by *Plasmodium* inside RBCs, it is unlikely that the lack of antimalarial activity observed in the IBSM study was due to differences in drug levels.

**Figure 5:**
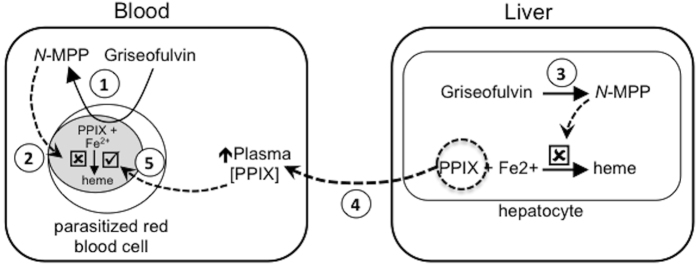
Hypothesized interaction of griseofulvin with parasitized red cells and the host. When *Plasmodium* parasitized red blood cells are exposed to griseofulvin, the drug induces the parasite to produce *N*-MPP (1). In the absence of PPIX (i.e. *in vitro* culture conditions), *N*-MPP inhibits ferrochelatase-catalyzed formation of heme from PPIX and Fe^2+^, and thereby prevents parasite growth (2). When griseofulvin is administered *in vivo*, the drug induces the formation of *N*-MPP in the liver (3). This results in the inhibition of hepatocyte ferrochelatase and a consequent build up of PPIX in the liver, which is released into the bloodstream (4). Circulating PPIX enters parasitized cells and out-competes the *N*-MPP inhibition of parasite-localized ferrochelatase. This allows ferrochelatase to continue producing heme and parasite growth to occur (5).

A second and more plausible reason for the lack of activity of griseofulvin in the clinical trial is that the griseofulvin treatment induced the accumulation of PPIX in the bloodstream. It is recognized that griseofulvin is metabolized in the liver of animals and humans to produce *N*-MPP. This inhibits the activity of hepatocyte FECH, causing the buildup of heme biosynthetic precursors, especially PPIX because FECH catalyzes the rate-limiting step. Griseofulvin treatment in humans elevates blood PPIX levels^21^,^22^ and we quantified the presence of PPIX in the plasma of the two IBSM subjects examined. Notably, the relative molar concentration of plasma PPIX and griseofulvin were comparable to that required to block the drug’s antimalarial activity in the *in vitro* growth assays (Table 1). Therefore, it is reasonable to hypothesize that the plasma PPIX produced during griseofulvin treatment was sufficient to block the antimalarial effect of griseofulvin *in vivo*. This hypothesis is depicted in Fig. 5.

In conclusion, griseofulvin represents a novel compound with antimalarial activity. After accumulating inside RBCs, griseofulvin impairs intraerythrocytic parasite growth by inhibiting FECH. The observation that parasite-encoded FECH is dispensable^9^,^10^, and instead that host RBC-derived FECH is required for intraerthrocytic development^8^, supports the hypothesis that griseofulvin acts against the parasite in a host-directed manner. However, the failure to translate the *in vitro* activity into a clinical trial indicates griseofulvin is not a promising antimalarial drug.

## Methods

### *P. falciparum* culture

*P. falciparum* strains 3D7 and K1 were cultured in purified human RBCs provided by the Australian Red Cross Blood Service, or from volunteers using standard methods^23^. The cell culture medium (CCM) comprised RPMI 1640 medium supplemented with 1X Glutamax, 0.2% Albumax (all from Life Technologies, Australia), 4% pooled AB + human serum (Invitrogen), 10 mM D-glucose, 25 mg/ml gentamycin, 6 mM HEPES and 0.2 mM hypoxanthine (all from Sigma Aldrich, Castle Hill, Australia). CCM lacking serum and Albumax (CCM-wash) was used for the cell washing.

### *In vitro* parasite growth assays

Griseofulvin (Sigma Aldrich, Castle Hill, Australia) was prepared as a 100 mM stock solution in dimethyl sulfoxide. In preliminary experiments to test griseofulvin anti-plasmodial activity, the compound was added directly to unsynchronized cultures of *P. falciparum* (to various final concentrations up to 200 μM) and the growth of parasites determined after the cultures were incubated for approximately 48 hours. To pretreat RBCs with griseofulvin, the compound was added to aliquots of uninfected RBCs (3% hematocrit in CCM) at specified concentrations and incubated at 37 °C. For the drug uptake assay experiments, cells were incubated in griseofulvin for between one and five days. For the *in vitro* parasite growth assays, cells were incubated with griseofulvin for three days, prior to the addition of trophozoite stage *P. falciparum* parasites prepared using percoll^24^ and added to the griseofulvin-treated RBCs at a final percentage parasitemia of 1%. Griseofulvin was also present in the cultures during the growth assays. For growth assays in the presence of PPIX, PPIX (Frontier Scientific, Logan, UT) was added to parasite cultures from a 100 mM stock solution prepared in dimethyl sulfoxide and protected from light. To study the parasite growth inhibition of blood samples from subjects who took griseofulvin in the *ex vivo* clinical trial, RBCs prepared from subject blood samples were infected with trophozoites and assayed for parasite growth in an identical manner, except that no griseofulvin was added to the parasite cultures. For all of the growth assays, control RBCs (either incubated in CCM without griseofulvin or collected from subjects before drug treatment commenced) were infected simultaneously with the same synchronized parasite preparations. Parasitemia and parasite developmental stages were determined by microscopic examination of Giemsa-stained thin blood smears (one smear for each culture replicate). Percentage parasitemia was calculated from the proportion of parasitized cells counted in at least 500 cells on each smear, and an additional 100 infected cells were scored for parasite developmental stage. Percentage growth inhibition was calculated from the proportion of parasitemia in treated versus control samples after subtracting the parasitemia of the cultures at the start of the assay.

### Determination of porphyrin levels in griseofulvin-treated parasites and plasma

RBCs in CCM (3% haematocrit and 30 ml medium) were preincubated with griseofulvin (100 μM) at 37 °C for three days. Ten-μl of percoll synchronized trophozoite stage parasites were added to obtain approximately 1% parasitemia. Parasites were cultured in CCM and griseofulvin in the dark for 48 h. An additional 10 μl synchronized trophozoite parasites was then added along with fresh CCM and griseofulvin and incubated for a further 72 h. Two control cultures were maintained under identical conditions: infected RBC not treated with griseofulvin, and uninfected RBC treated with griseofulvin. The RBC in treated and control cultures were harvested by centrifuging at 500 *g* for 10 min at 24 °C. Cellular and plasma porphyrins were extracted and analysed as described in Supplementary Methods.

### Determination of griseofulvin levels in plasma and RBCs

Griseofulvin concentrations in drug-treated cultured RBCs, and blood (plasma and RBCs) from subjects who participated in the *ex vivo* and IBSM clinical studies, were determined with a validated method using liquid chromatography-tandem mass spectrometry (LC-MS/MS), as described in Supplementary Methods.

### *Ex vivo* study to assess the effect of griseofulvin in parasite growth

We conducted an exploratory clinical study at Macquarie University (Sydney, Australia). The aim of the study was to investigate if the blood of subjects who had taken griseofulvin inhibited the growth of cultured *P. falciparum*. Malaria naïve healthy volunteers 35–60 years of age who met the inclusion and exclusion criteria were eligible for the study (see study protocol in Supplementary Material). Subjects were treated with griseofulvin (Grisovin^®^ 500 mg tablets, Aspen, Australia) with a meal at a dose of 1,000 per day (500 mg bi-daily [BD] each 12 hours for 4 days) or a single 2,000 mg dose. Blood samples were collected into 5 ml sodium citrate tubes (Becton Dickinson, Australia) before griseofulvin treatment (no treatment control) and then once daily for up to four days of griseofulvin treatment. Additionally, griseofulvin levels were measured in all subjects 12, 14 or 15 days after the first griseofulvin dose. Samples were centrifuged at 170 *g* for 13 minutes and the plasma and RBC fractions collected into separate tubes; white cell fractions were discarded. The plasma fraction was centrifuged at 5000 *g* for 5 min and the clarified supernatant removed and kept at −20 °C for plasma analysis. RBCs were washed twice in CCM-wash (ten volumes) and stored at 4 °C, and used to determine parasite growth within three days of collection. Separate samples of purified RBCs were stored at −20 °C for griseofulvin quantification. Parasite growth in the purified RBCs was determined using the *in vitro* parasite growth assay method described above. As there were no available data to calculate sample size, following ethical and safety guidelines the study was restricted to a pilot exploratory study including three subjects taking 1,000 mg/day (500 mg BD) for four days, and one additional subject taking a single 2,000 mg dose.

### IBSM study to assess the effect of griseofulvin in parasite growth

A pilot phase I, single center, open label, randomized, controlled clinical trial was undertaken at Q-Pharm Pty Ltd (Brisbane, Australia). The study comprised two cohorts. The primary objective of the first cohort was to characterize the pharmacokinetic-pharmacodynamic relationship of griseofulvin on clearance of *P. falciparum* in healthy subjects following infection with blood stage parasites. The primary objective of the second cohort was to investigate the prophylactic activity of griseofulvin on growth of blood stage *P. falciparum* in healthy subjects. The secondary objective of both cohorts was to assess the tolerability of griseofulvin. The infectivity control group for Cohort 2 included subjects from another malaria clinical trial carried out simultaneously in the same clinical unit using an identical inoculum. The results from the control group will be published elsewhere. Healthy male and female subjects 18–55 years of age who met the inclusion and exclusion criteria (see study protocol in Supplementary Material) were eligible for the study.

IBSM infection was performed as previously described^18^. Briefly, subjects were inoculated on Day 0 intravenously with ~1,800 viable *P. falciparum*-infected human erythrocytes of the chloroquine-sensitive 3D7 strain. Parasite growth was monitored by quantitative PCR (qPCR) targeting *P. falciparum* 18S rDNA as described previously^25^. In Cohort 1, a statistician created a random sequence of subjects from computer-generated random numbers, which was used to allocate subjects in a 4:1 ratio to griseofulvin (Grisovin^®^ 500 Tablets, Aspen, Australia) or mefloquine (Lariam^®^, Roche, Australia). Subjects in the griseofulvin arm of Cohort 1 were to receive griseofulvin in a dose of 500 mg each 12 hours. Griseofulvin was administered after a meal from Day 6 to up to Day 9. Control subject in Cohort 1 received a single dose of mefloquine (10 mg/kg) when parasitemia reached designated levels (≥1,000 parasites/ml). All subjects in Cohort 1 were to receive curative treatment with artemether-lumefantrine (Riamet^®^, Novartis Pharmaceuticals Pty Ltd, Australia) on Day 20 or earlier if parasitemia failed to clear, or if recrudescence occurred. Subjects in Cohort 2 were scheduled to receive the same dose of griseofulvin as Cohort 1 from Day -4 (i.e., 4 days before inoculation) until Day 6. Control subjects in Cohort 2, who participated in another study undertaken simultaneously, received their designated study drug on Day 7 as described in the protocol of that study. Subjects in the griseofulvin arm of Cohort 2 were to receive rescue treatment with artemether-lumefantrine when all subjects reach the designated parasitemia levels (≥1,000 parasites/ml).

The primary efficacy endpoint for Cohort 1 was the PRR as determined by qPCR kinetics of parasite clearance^26^. Parasitemia was measured on Day 0 (pre-inoculation), Day 4 (AM), twice daily (AM and PM) from Day 5 to Day 12, on Day 13 and Day 14 (AM) and at the end of study visit (Day 28). The primary efficacy endpoint for Cohort 2 was the PMR estimated by linear regression of the log-transformed parasite concentrations measured by qPCR. Parasitemia was measured on Day 0 (pre-inoculation), Day 4 (AM), twice daily (AM and PM) from Day 5 to Day 7, and on Days 8, 9 and 13, and at the end of study visit (Day 28).

Griseofulvin levels were measured twice daily (AM and PM) from Day 6 to Day 10 for Cohort 1, and once daily on Days -4, 0, 7 and 9 for Cohort 2.

Eight subjects in a dose cohort have proven sufficient to characterize the effect of a drug on malaria parasite kinetics after infection with IBSM of healthy subjects^18^. However, since pre-clinical data indicated an anti-plasmodial effect for griseofulvin, it was decided to include only four experimental subjects in Cohort 1. We postulated that if no effect was seen in four subjects, it was unlikely that an effect would be seen in a larger cohort. In Cohort 2 it was planned to enroll six subjects.

### Safety assessment

Safety assessment was conducted in both clinical trials described in this report. For the *ex vivo* study, adverse effects assessment included blood sampling of study subjects for complete blood cell count, and electrolyte and liver function enzymes analyses. For the IBSM study, safety parameters included physical examination, vital signs, ECGs and clinical laboratory tests. Adverse events were monitored during the study via telephone or in the clinical unit. Safety was determined by recorded adverse events, which were categorized according to their causality (probable, possible or unlikely related, or unrelated to study treatments) and severity (mild, moderate or severe), and whether they were serious or non-serious.

### Statistical analysis

Means, standard deviation (SD), standard error of the mean (SEM) and statistical testing were determined using Excel and GraphPad Prism 6. In the IBSM study, STATA version 13 was used to calculate peak plasma values, range, and coefficient of variation (CV%); ANOVA was used to compare peak griseofulvin levels between cohorts.

### Ethics

Ethical approval to work with cells for the *in vitro P. falciparum* growth inhibition assays was obtained from the Macquarie University and Australian National University Human Research Ethics Committees (project number 5201200714 and 2014/732, respectively). The *ex vivo* parasite growth inhibition assay study was approved by the Macquarie University and Australian National University Human Research Ethics Committees (5201300061), and registered in the Australian and New Zealand Clinical Trials Registry (ANZCTRN12613000307707 date of registration 20/03/2013). The IBSM challenge study was approved by QIMR Berghofer Human Research Ethics Committee, Macquarie University and Australian National University Human Research Ethics Committees (P1536). The study was registered in the Australian and New Zealand Clinical Trials Registry (ANZCTRN12613000698774, date of registration 26/06/2013). The study undertaken in parallel with Cohort 2 of the griseofulvin malaria challenge study was registered at clinicaltrials.org (NCT02389348, date of registration 20/02/2015) and approved by QIMR Berghofer Human Research Ethics Committee. All the methods involving human subjects and material derived from human subjects were performed in accordance with the study protocols and institutional regulations. All subjects gave written informed consent before being included in the studies.

## Additional Information

**How to cite this article:** Smith, C. M. *et al*. Griseofulvin impairs intraerythrocytic growth of *Plasmodium falciparum* through ferrochelatase inhibition but lacks activity in an experimental human infection study. *Sci. Rep.* **7**, 41975; doi: 10.1038/srep41975 (2017).

**Publisher's note:** Springer Nature remains neutral with regard to jurisdictional claims in published maps and institutional affiliations.

## Supplementary Material

Supplementary Material

## Figures and Tables

**Table 1:** PPIX and griseofulvin concentrations used in the *in vitro* parasite growth inhibition experiments and determined in the IBSM study.

Study (subject sample)	[PPIX] (μM)	[Griseofulvin] (μg/L)	[Griseofulvin] (μM)	PPIX:Griseofulvin molar ratio
*In vitro*	0.16		50	1:313
*In vitro*	0.8		50	1:63
*In vitro*	1.6^b^		50	1:31
*In vitro*	4^b^		50	1:13
IBSM (I6)^a^	0.08^c^	767^d^	2.17	1:27
IBSM (I7)^a^	0.12^c^	747^d^	2.12	1:18

^a^Plasma sample collected on study Day 7 in IBSM Cohort 2.

^b^Complete blockade of griseofulvin parasiticidal activity observed at these PPIX concentrations (determined in Fig. 2b).

^c^PPIX concentrations determined by HPLC MS-MS (Supplementary Fig. S5).

^d^Plasma griseofulvin concentration (determined in Fig. 4c).

**Table 2:** Treatments administered to subjects in the *ex vivo* and IBSM studies.

Subject	Griseofulvin dose (mg) (number doses)^a^	Griseofulvin treatment duration (study day)	Commencement of artemether-lumefantrine treatment (study day)
***Ex vivo*** **study**			
E1-E3	500 (BD, 8)	Day 1 AM – Day 4 PM	NA
E4	2,000 (single dose)	Day 1	NA
**IBSM challenge study**			
I1	500 (BD, 7)	Day 6 AM – Day 9 AM	Day 9 PM
I2	500 (BD, 4)	Day 6 AM – Day 7 PM	Day 8 AM
I3^b^	500 (BD, 7)	Day 6 AM – Day 9 AM	Day 9 PM (IV artesunate)
I4^c^	Mefloquine (687 mg, single dose)	Day 8 AM	Day 12 PM
I5	500 (BD, 8)	Day 6 AM – Day 9 PM	Day 10 AM
I6 and I7	500 (BD, 22)	Day -4 AM – Day 6 PM	Day 7 AM

^a^BD: bi-daily dosage (AM and PM).

^b^Subject I3 was treated with IV artesunate (180 mg) on Day 9 PM at the hospital where he was admitted to treat a serious adverse event unrelated to the study (renal colic).

^c^Subject I4 was the control subject of IBSM Cohort 1 and was treated with mefloquine instead of griseofulvin. This subject also received treatment with artemether-lumefantrine.

**Table 3:** Griseofulvin levels in plasma and RBCs.

Study and cohort	Peak griseofulvin levels (μg/L)^a^ (range)^b^	Coefficient of variation (%)	p value^c^
Plasma			0.013
*ex vivo* study 1,000 mg dose Plasma	1,830 (1,776, 1,884)	3	
*ex vivo* study 2,000 mg dose Plasma	1,234	NA	
IBSM study Cohort 1 Plasma	1,378 (1,201, 1,588)	12.1	
IBSM study Cohort 2 Plasma	1,079 (821, 1,337)	33.8	
RBCs			0.21
*ex vivo* study 1,000 mg dose RBC	110 (59, 143)	40.9	
*ex vivo* study 2,000 mg dose RBC	63	NA	
IBSM study Cohort 1 RBC	149 (123, 184)	20.2	
IBSM study Cohort 2 RBC	86 (55, 117)	51.0	

^a^Peak griseofulvin levels are expressed as average.

^b^Range values for the 2,000 mg dose of the *ex vivo* study are not provided because only one subject was dosed.

^c^ANOVA was used to compare peak griseofulvin levels values between cohorts (p value was significant if p < 0.05). The 2,000 mg dose of the *ex vivo* study was not included in this analysis since this group had only one subject and therefore was not appropriate to be included in the analysis. NA, not analyzed.
