# Supplementary material for: Griseofulvin impairs intraerythrocytic growth of Plasmodium falciparum through ferrochelatase inhibition but lacks activity in an experimental human infection study
Source: Sci Rep. 2017 Feb 8;7:41975. doi: 10.1038/srep41975 (PMC5296727; doi:10.1038/srep41975)
Supplement: Supplementary Material [file srep41975-s1.pdf]

Griseofulvin impairs intraerythrocytic growth of *Plasmodium falciparum* through ferrochelatase inhibition but lacks activity in an experimental human infection study.

Clare M Smith[1#], Ante Jerkovic[2], Thy Thuc Truong[3], Simon J Foote [4*], James S McCarthy[5*],and Brendan J McMorran[4*]

1. School of Medicine and The Menzies Research Institute, University of Tasmania, Hobart, Tasmania, Australia.
2. Department of Biomedical Sciences, Faculty of Medicine and Health Sciences, Macquarie University, Sydney, New South Wales, Australia.
3. Joint Mass Spectrometry Facility, Research School of Chemistry, The Australian National University, Canberra, Australian Capital Territory, Australia.
4. The John Curtin School of Medical Research, The Australian National University, Canberra, Australian Capital Territory, Australia.
5. QIMR Berghofer Medical Research Institute, Brisbane, Queensland, Australia

Department of Microbiology and Physiological Systems, University of Massachusetts Medical School, Worcester, MA 01605, USA

* Joint senior authors: Simon J Foote, James S McCarthy and Brendan J McMorran.

Corresponding author: Brendan J McMorran.

Supplementary Methods.

Determination of porphyrin levels in griseofulvin-treated parasites and plasma samples – porphyrin extraction and analysis.

RBCs were lysed in 1 ml of 0.2% acetic acid followed by gentle vortexing for ~20 s. To extract the free *N*-MPP and other porphyrins, 100% ethanol (48 ml) was added to the lysed cells and the mixture shaken vigorously for ~20 s. The samples were then centrifuged at 3000*g* for 15 min at 24°C. The supernatant was collected into fresh 15 ml plastic tubes and volumes were reduced to approximately 3 ml using vacuum centrifugation. The concentrated samples were reconstituted to 15 ml with 0.2% acetic acid in Milli-Q water, followed by C-18 solid phase extraction (SPE) purification and concentration as previously described [1]. Samples were eluted with 100% methanol (1.5 ml) into 1.5-ml plastic tubes, dried by vacuum centrifugation, and resuspended in 100 µL of 100% methanol with treatment in an ultrasonic bath for 5 min. The dissolved porphyrins were centrifuged at 16,000*g* for 10 min prior to UHPLC analysis. UHPLC and UV-absorption spectroscopy were used to detect and quantify porphyrin concentrations, according to the method described in Benton *et al.* [2]with some modifications [1]. Concentrations of *N*-MPP were determined by comparison to a separately prepared standard curve using *N*-MPP (Frontier Scientific, Logan, UT; concentration range 25-1,000 nM).

To determine porphyrin levels in plasma, samples (0.1 ml) were added to 10 volumes of 100% ethanol, concentrated using a SpeedVac concentrator (Thermo Savant) at 45°C, and then resuspended in 0.2% acetic acid in Milli-Q water. Samples (7 µL) were injected onto an Agilent Zorbax Eclipse 1.8 µm XDB-C18 2.1 × 50 mm column. Solvent A consisted of 0.1 % aqueous formic acid and solvent B of methanol with 0.1 % formic acid. The porphyrins were eluted with a linear gradient from 10-35 % solvent B over 3 min, 35-90 % solvent B from 3-9 min (then held at 90 % from 9-14 min) at a flow rate of 200 µl/min. The total analysis time was 30 min. The column effluent was analysed by an Agilent 6530 Accurate Mass LC/MS QTOF with an electrospray

interface (ESI) Jetstream ion source interface (Agilent Technologies, Inc., Santa Clara, CA, USA). The positive ion polarity parameters were: gas temperature 250°C, drying gas flow 7 L/min, nebuliser 35 psig, sheath gas temperature 325°C and sheath gas flow 11 L/min, capillary voltage 3500 V, nozzle voltage 500 V and fragmentor voltage 125 V. The QTOF was operated in the extended dynamic range mode and data acquired using targeted MS/MS with a m/z 1.3 isolation window prior to collision induced dissociation (CID; N_2 collision gas supplied at 18 psi). Mass spectra were acquired at 2 spectra/s and MS/MS at 3 spectra/s over a range of m/z 50-1,000. Data were acquired and analysed using the Agilent Technologies MassHunter software (ver. B.5.0). A three point 6-log standard curve was generated using PPIX ((Frontier Scientific, Logan, UT).

Determination of griseofulvin levels in plasma and RBCs

Plasma and RBC samples were precipitated with three volume equivalents of a mixture of acetonitrile with 1% formic acid containing the internal standard griseofulvin-^{13}C,D3 (Toronto Research Chemicals, Inc). After protein precipitation, samples were centrifuged and 3 µl were injected into a UPLC column (Acquity UPLC BEH C_{18}, 2.1 x 50 mm, particle size 1.7 µm) maintained at 45°C. Solvent A consisted of 2 mM ammonium acetate in water + 0.1% formic acid, and solvent B of 2 mM ammonium acetate in methanol + 0.1% formic acid. The flow rate was kept at 0.4 ml/min. An initial isocratic method was applied (50% solvent B). Samples were separated with the following gradient: 0 min 50 % solvent B, 1.2 min 65% solvent B, 1.21 min 98% solvent B, 1.7 50% solvent B. The total analysis time was 2 min. The separated compounds were detected with an ESI ion source in positive ion mode (Premier XE Waters Corporation, USA). The specific time-points at which griseofulvin was determined are specified in the sections describing the clinical studies.

Supplementary Methods References

1 Smith, C. M. *et al.* Red cells from ferrochelatase-deficient erythropoietic protoporphyria patients are resistant to growth of malarial parasites. *Blood* **125**, 534-541, doi:10.1182/blood-2014-04-567149 (2015).

2 Benton, C. M., Lim, C. K., Moniz, C. & Jones, D. J. Ultra high-performance liquid chromatography of porphyrins in clinical materials: column and mobile phase selection and optimisation. *Biomedical chromatography : BMC* **26**, 714-719, doi:10.1002/bmc.1720 (2012).

Supplementary Figure S1.

Supplementary Figure S1. Levels of griseofulvin measured in red blood cells incubated in different drug concentrations for different periods of time. Assays were performed once for each indicated concentration and time point.

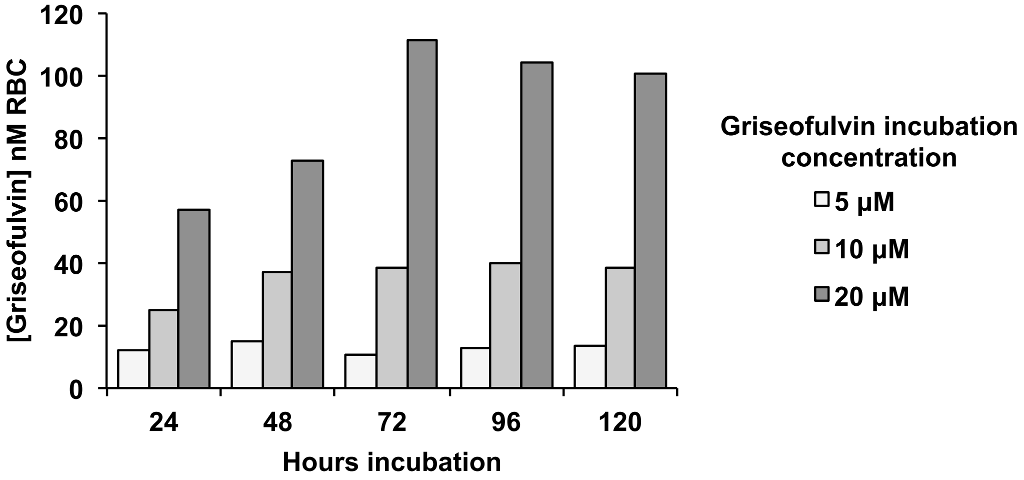

Supplementary Figure S2

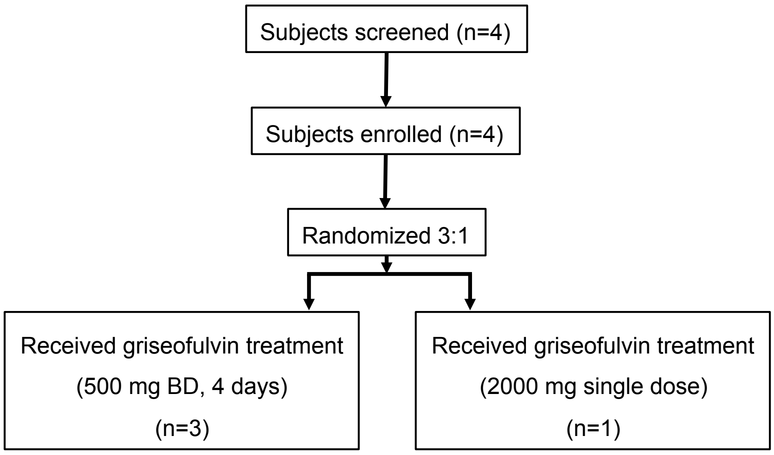

Supplementary Figure S2. ***Ex vivo*** **griseofulvin clinical study protocol flow chart**. All subjects completed the study and were included in the analysis. No control subjects were enrolled.

Supplementary Figure S3

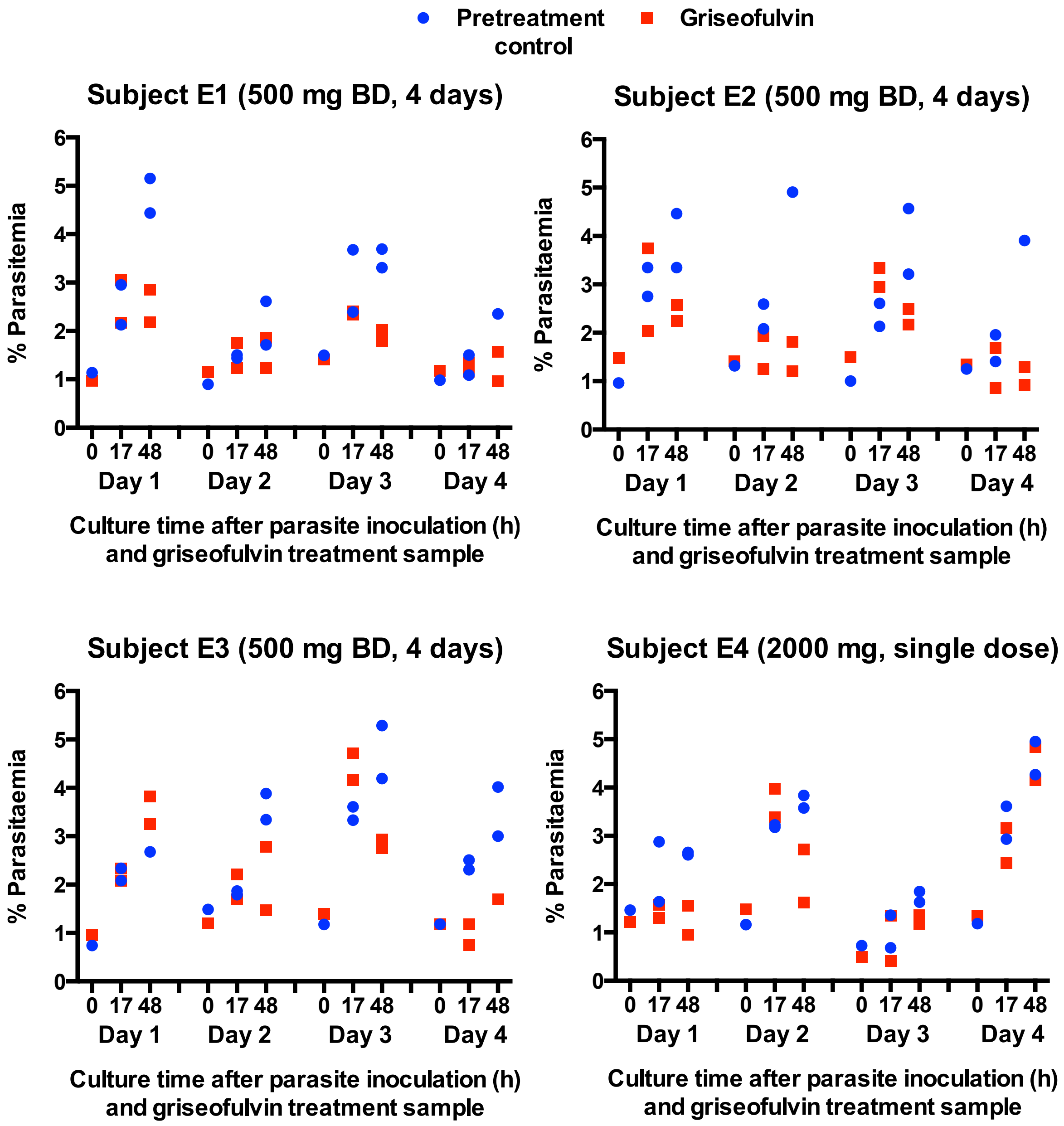

Supplementary Figure S3. Numbers of parasitized cells counted in the *ex vivo* study. Percentage parasitemias were determined in parasite cultures immediately following, and after 17 and 48 h incubation, inoculation of purified trophozoite stage parasites into RBCs collected from subjects receiving 500 mg griseofulvin BD (E1-3), one subject receiving a single 2000 mg dose (E4), samples collected before commencement of the treatment (pretreatment control). Each treatment sample (Day 1-4) was assayed separately by infecting treatment and control RBCs simultaneously with the same parasite inoculum; duplicate cultures were established for the 17 and 48 h time points.

Supplementary Figure S4

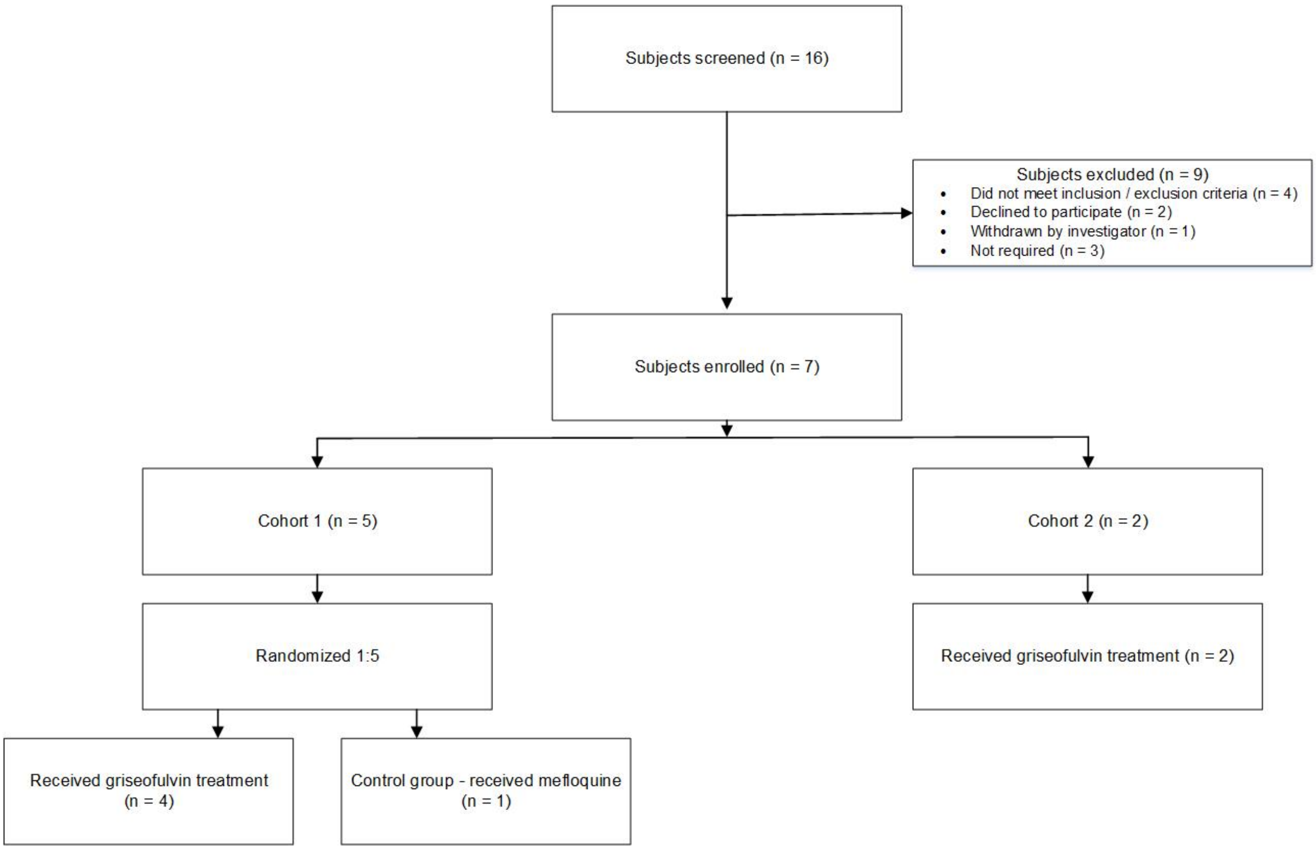

Supplementary Figure S4. IBSM clinical study protocol flow chart. All subjects completed the study and were included in the analysis. No control subjects were enrolled for Cohort 2. A cohort (n=8) from another study undertaken simultaneously in the same clinical unit, which was inoculated with the same inoculum, was used as a control group.

Supplementary Figure S5.

A **MS-MS product ion spectrum of the PPIX parent ion (M+H: 563.26)**

	MF	MW	[M+H]+	Product ions (most to least intense)	RT (min)
PP IX	C34 H34 N4 O4	562.2660	563.2653	445.2340, 431.2176, 489.2248, 504.2458, 430.2105	15.2

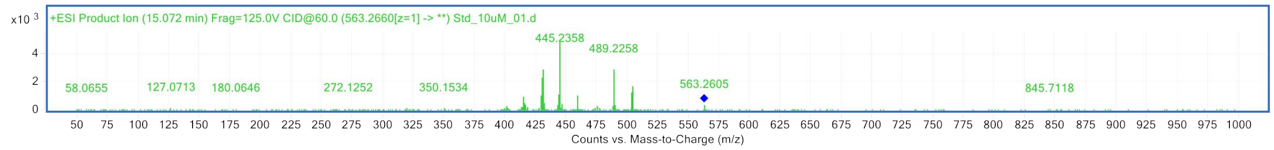

B **MS-MS spectra of PPIX product ion (445.23) in PPIX standards (added to normal plasma).**

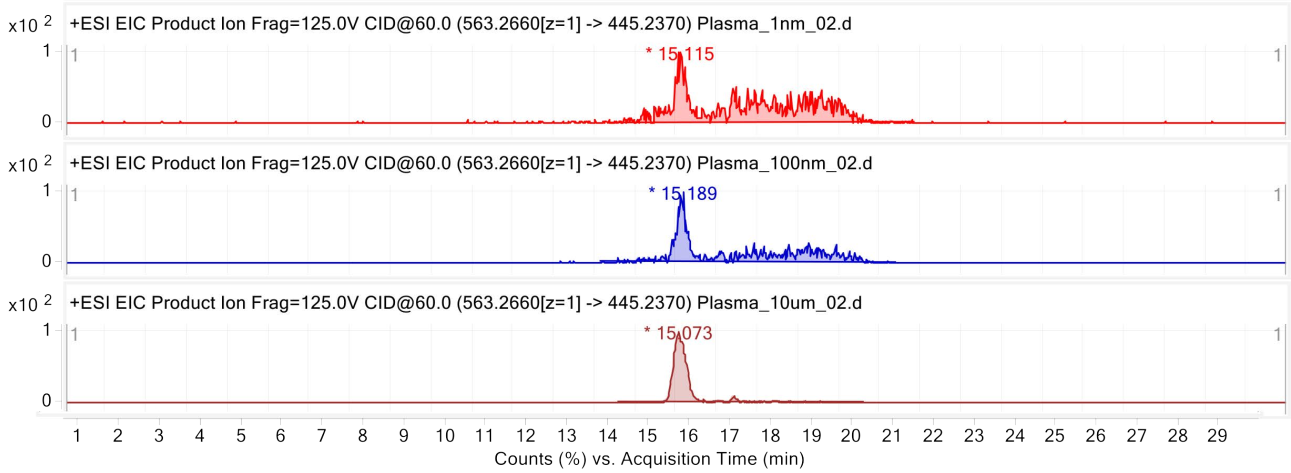

C

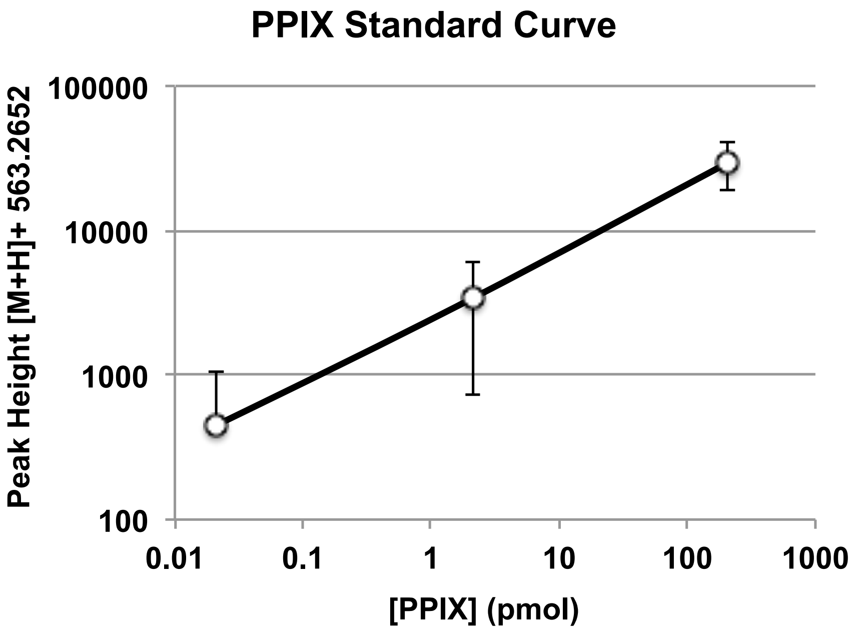

Supplementary Figure S5 (continued)

D

	MF	MW	[M+H]+	Product ions (most to least intense)	RT (min)
PP IX	C34 H34 N4 O4	562.258	563.2653	445.2320, 431.2155, 489.2211, 504.2434, 430.2083	17.3

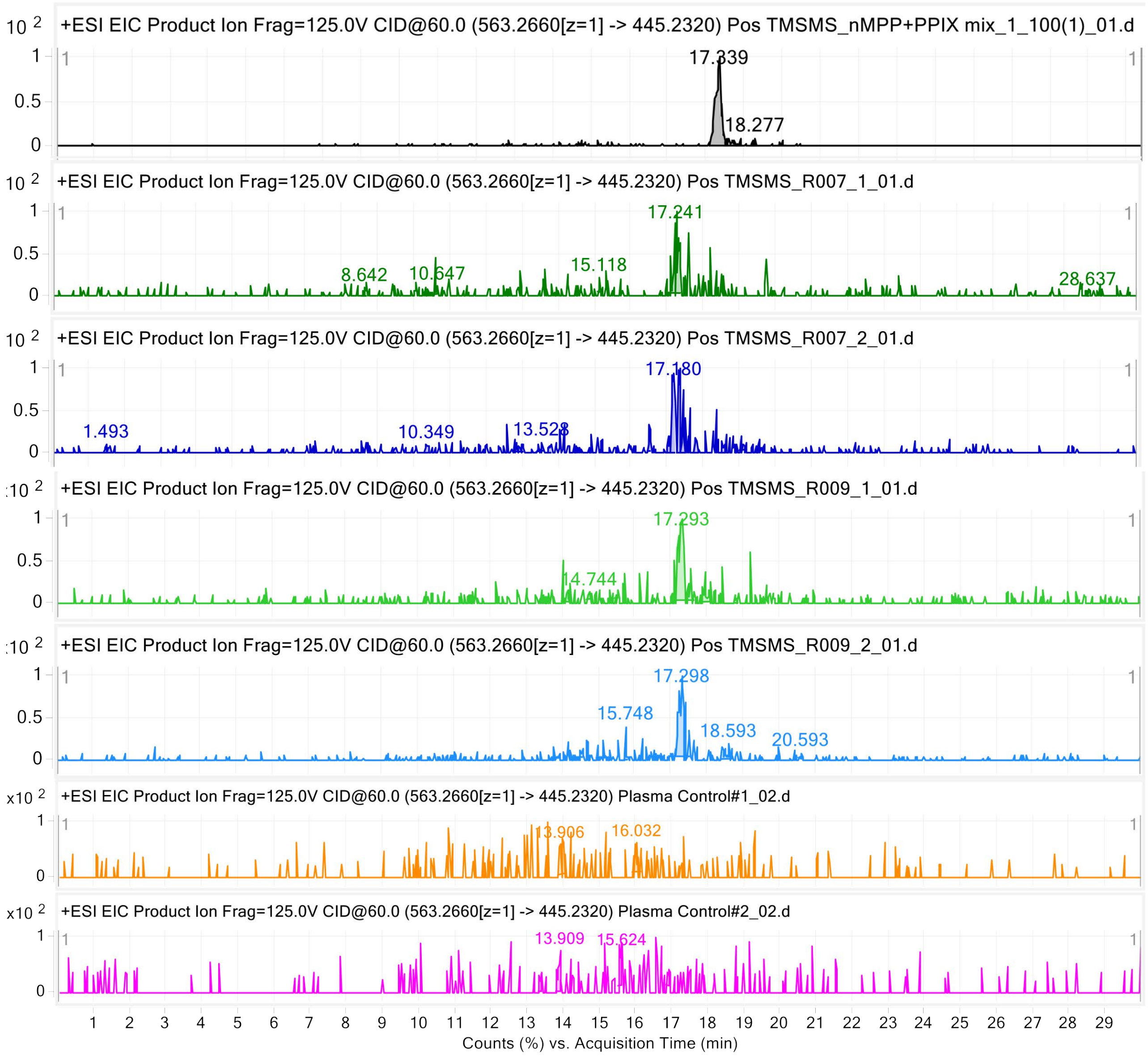

MS-MS spectra of PPIX product ion (445.23) in plasma from two griseofulvin-treated IBSM volunteers I6 (R007)) and I7 (R009) (2 replicates each), and two plasma samples from untreated individuals (control 1 and 2). Note the retention time differs from that in Supplementary Figure S5B.

Supplementary Figure S5 (continued)

E

Plasma sample	replicate	Peak height	PPIX (pmol)	Plasma [PPIX] (μM)
R007	1	93	0.000732	0.052
	2	125	0.001400	0.100
	3	127	0.001450	0.104
	4	88	0.000649	0.046
	**mean**			**0.076**
	SD			*0.030*
R009	1	96	0.000785	0.056
	2	158	0.002330	0.166
	3	161	0.002430	0.174
	4	125	0.001400	0.100
	**mean**			**0.124**
	SD			*0.056*
Control 1		ND		
Control 2		ND		

MS-MS spectra peak heights, PPIX amount determined using the standard curve (C) and PPIX plasma concentrations. SD, standard deviation; ND, no peak detected.

Supplementary Table S1. Demographic profile of randomised subjects in the IBSM study

Age (years)	Mean ± SD (range)	27.4 ±11.2 (18-52)
Age groups	18 - 30	6 (85.71%)
	>45	1 (14.29%)
Sex	Male	7 (100.00%)
Race	Caucasian	5 (71.43%)
	Asian	2 (28.57%)
BMI	Mean ± SD (range)	23.9 ±2.5 (19.3-26.8)
Height (cm)	Mean ± SD (range)	178.0 ±4.4 (171-183.0)
Weight (kg)	Mean ± SD (range)	76.2±11.2 (56.5-86.8)

Supplementary Table S2. Common adverse events in the IBSM study.

Adverse Event	Number reported
Headache [a]	10
Fatigue	4
Nausea	4
Vomiting	4
Fever	3
Myalgia	3
Renal colic [b]	1

[a] One mild adverse event (headache) was reported as possible related to griseofulvin.

[b] Classified as serious, but not related to trial study.
